# Transition from sea to land: olfactory function and constraints in the terrestrial hermit crab *Coenobita clypeatus*

**DOI:** 10.1098/rspb.2012.0596

**Published:** 2012-06-06

**Authors:** Anna-Sara Krång, Markus Knaden, Kathrin Steck, Bill S. Hansson

**Affiliations:** Department of Evolutionary Neuroethology, Max Planck Institute for Chemical Ecology, Hans-Knöll-Strasse 8, 07745 Jena, Germany

**Keywords:** olfaction, olfactory system, adaptations, terrestrial crustaceans, odourants

## Abstract

The ability to identify chemical cues in the environment is essential to most animals. Apart from marine larval stages, anomuran land hermit crabs (*Coenobita*) have evolved different degrees of terrestriality, and thus represent an excellent opportunity to investigate adaptations of the olfactory system needed for a successful transition from aquatic to terrestrial life. Although superb processing capacities of the central olfactory system have been indicated in *Coenobita* and their olfactory system evidently is functional on land, virtually nothing was known about what type of odourants are detected. Here, we used electroantennogram (EAG) recordings in *Coenobita clypeatus* and established the olfactory response spectrum. Interestingly, different chemical groups elicited EAG responses of opposite polarity, which also appeared for *Coenobita compressus* and the closely related marine hermit crab *Pagurus bernhardus.* Furthermore, in a two-choice bioassay with *C. clypeatus,* we found that water vapour was critical for natural and synthetic odourants to induce attraction or repulsion. Strikingly, also the physiological response was found much greater at higher humidity in *C. clypeatus*, whereas no such effect appeared in the terrestrial vinegar fly *Drosophila melanogaster*. In conclusion, our results reveal that the *Coenobita* olfactory system is restricted to a limited number of water-soluble odourants, and that high humidity is most critical for its function.

## 1. Introduction

The ability to recognize and respond to chemical cues in the environment is essential in most animals. In crustaceans, chemosensory cues are used to detect and assess food, mating partners, predators, competitors and suitable habitats, and for communication with conspecifics (see [1] and references therein). Adaptation to terrestrial life has evolved independently in at least five major crustacean lineages [2,3]. One of these, the anomuran Coenobitidae, comprises the land hermit crabs, *Coenobita* spp. (15 species) and the closely related robber crab *Birgus latro* [4]. A successful transition from aquatic to terrestrial life requires specific adaptations and raises dramatically new demands on the olfactory system of an animal [2,4,5]. While odour stimuli in aquatic environments are mainly hydrophilic, polar molecules, odourants on land are often hydrophobic and need to be volatile (discussed in the study of Hansson *et al*. [5]). Hence, these land crabs represent an excellent opportunity to investigate the effects of the transition from sea to land on the olfactory system.

Since the Coenobitidae first emerged on land *ca* 20 Ma (oldest fossils dated to the Lower Miocene) [6], they have evolved an olfactory system that is functional on land. Behavioural studies have shown that they use olfaction to localize potential, distant food items [7–9]. *Coenobita* have even been shown to prefer odours from food not recently eaten [8], to locate shells through odours of dead conspecifics [10] and to discriminate between odours from well water and seawater [11]. While other terrestrial crustaceans investigated, such as isopoda Oniscoidae (wood lice), show indications of greatly reduced olfactory systems, with minute-sized antennules and diminished or even lacking olfactory lobes [12], neuroanatomical studies on the central olfactory system in *Coenobita clypeatus* and *B. latro* instead indicate superb processing capacities [13–15].

To detect and orient to distant chemical cues, crustaceans use specialized sensilla, aesthetascs, localized on the lateral flagellum of the antennules (first antennae) [16,17], whereas several types of bimodal sensilla containing chemo- and mechanoreceptor neurons on the antennules and other parts of the body are mainly used for close range/contact evaluation, but may also be involved in food search [17,18]. Morphological antennular investigations in *Coenobita compressus* [19] and *B. latro* [9] have indicated an adaptation for function in air, with aesthetascs being short and blunt, and structurally different from the long and slender aesthetascs found in aquatic decapods. Functional evidence of the antennules as olfactory organs has hitherto been lacking in *Coenobita*, but has been shown for *B. latro* [9]. Using electroantennogram (EAG) recording techniques, Stensmyr *et al*. [9] showed that *B. latro* antennules respond in a sensitive and selective manner to carbon dioxide, water vapour, as well as to volatile odours from food items and a few selected synthetic odourants. The only other terrestrial crustacean where physiological responses to airborne odour stimuli have been investigated is the fully terrestrial desert isopod *Hemilepistus reaumuri* [20].

Here, we combine electrophysiological and behavioural approaches to reveal biologically active odourants in the terrestrial hermit crab *C. clypeatus*. We present the first comprehensive EAG-based screen for odourant physiological activity, including dose–response curves for some of the active odourants, and establish the behavioural significance of some food blends and individual compounds in a two-choice bioassay. We furthermore compare the EAG responses of *C. clypeatus* to those of the congeneric terrestrial *C. compressus* and the closely related marine hermit crab *Pagurus bernhardus*. Finally, we investigate the humidity dependency of the olfactory sense in *C. clypeatus* and compare the effect of humidity on EAG responses with those of the vinegar fly *D. melanogaster,* representing a more distantly related arthropod with a much longer evolutionary history on land.

## 2. Material and methods

### (a) Animals

*Coenobita clypeatus* (Herbst) originating from the Caribbean were obtained from a local animal supplier (Import-Export Peter Hoch GmbH, Waldkirch, Germany). They were kept in glass terraria (60 × 30 × 30 cm) at 20–26°C, more than 60 per cent humidity, with moist sand substrate (*ca* 8 cm depth), shelters, surplus of shells of different sizes, access to distilled water and artificial seawater (ASW) as drinking water, and a variety of food. Adult males and females (shell size 25–44 mm Ø) were used in the different experiments regardless of sex. *Coenobita compressus* (H. Milne Edwards) originating from Ecuador was obtained from a local animal supplier (Simontorp Säteri AB, Blentarp, Sweden) and were reared similar to *C. clypeatus. Pagurus bernhardus* (L.) from the North Sea was obtained from the Biological Institute Helgoland and kept in ASW at 12.5–13°C and fed crab food (TetraCrusta Menu, Tetra, Melle, Germany). Experiments with vinegar flies *D. melanogaster* were carried out on 4–6 days old, mated females of the Canton-Strain. Fly cultures were maintained under a 12 L : 12 D cycle at 25°C and 65 per cent humidity, on a standard cornmeal medium.

### (b) Synthetic odourants

Synthetic compounds were obtained from Sigma-Aldrich, Fluka or Merck at highest available purity. The following compounds were used as solutions in water: ammonium hydroxide 32 per cent, trimethylamine 40–45 per cent, ethylamine 70 per cent and dl-lactic acid 85 per cent. Cis-3-hexenal was used as 50 per cent solution in triacetin. For the EAG experiments, compounds were diluted in hexane, except for trimethylamine and benzyl alcohol that were dissolved in ethanol; l-(+)-lactic acid and all amino acids that were dissolved in distilled water; and cresol, phenol, furfural, benzoic acid, menthol and *γ*-butyrolactone that were dissolved in dichloromethane. For the bioassays, all synthetic compounds as well as the banana extract (see §2*d*), were diluted 10^−2^ in mineral oil (pro-ultra, for molecular biology, Sigma-Aldrich).

### (c) Electroantennogram

In order to identify physiologically active compounds in *C. clypeatus*, a broad EAG screening was made, using a total of 140 compounds from different chemical classes, including acids, aldehydes, amines, alcohols, esters, lactones, ketones, aromatics, cyclic terpenes, ethers, furans, thiocyanates, amino acids and the solvents. Additionally, water vapour and carbon dioxide were tested. EAG recordings were performed according to Stensmyr *et al*. [9] (for details, see electronic supplementary material). All compounds were tested on at least five antennular preparations. Compounds that elicited activity in at least two out of five preparations were tested further until a minimum of 10 antennular preparations had been tested (i.e. *n* ≥ 5 for compounds scored as inactive, *n* ≥ 10 for compounds scored as active). Compounds were defined as active if they elicited an average response greater than 30 mV or less than or equal to −30 mV.

Dose–response curves were established for some physiologically active compounds in *C. clypeatus*. These compounds were presented in ascending steps from 1 : 1000 (10^−3^) or 1 : 500 dilutions, up to 10^−1^, using the same procedure and set up as for the screening. Furthermore, for comparison, similar dose–response curves were established for *C. compressus*, and a few of these compounds were tested for response also in the closely related marine hermit crab *P. bernhardus*.

To investigate how humidity affects the physiological response in *C. clypeatus*, odourants (dilutions from 10^−4^ to 10^−1^) were tested for EAG activity at high and low humidity levels on five antennular preparations (*n* = 5). High humidity (86–91% relative humidity (RH)) was obtained by increasing the humidified, continuous airstream, while decreasing the stimuli/compensatory airflow, and at the same time heating the water within the gas-washing bottle. At the lower humidity level, the humidifying step was omitted, thus leaving the airstream at room RH levels (32–35% RH; for details, see electronic supplementary material). In another set of experiments, several compounds defined as physiologically inactive in the initial screening experiment were re-tested at 10^−1^ dilutions at the higher humidity level (*n* = 5).

For comparison, EAG recordings were performed at different humidities under identical conditions as described for *C. clypeatus*, using the vinegar fly *D. melanogaster* presenting odourants at 10^−1^ dilutions (*n* = 6; for details, see electronic supplementary material).

### (d) Bioassay

A two-choice bioassay was conducted with *C. clypeatus*, using 20 test arenas (plastic boxes, 52 × 43 × 28 cm) with a centrally placed shelter and one pit-fall on each short side (for details, see electronic supplementary material). Crabs were placed individually in the boxes. During a 24 h acclimatization period, crabs had no access to food or water, and lids covered the pit-falls to prevent the crabs from entering. At the start of the experiment, a test odourant (water, food item or chemical compound; see below) was added into a small plastic cup (4 cm height, 4 cm Ø), which was placed into either of the two pit-falls, and a control cup was placed into the second pit-fall. Thereafter, the lids covering the pit-falls were removed and experiments ran overnight since in *Coenobita*, activity is largely nocturnal [4]. The following morning, the position of the crab was noted. Each test odourant was tested over at least two nights until a minimum of 26 crabs had entered a pit-fall (i.e. *n* ≥ 26).

In a pilot test, it was noted that water deprivation was critical to motivate food search, since the crabs seldom entered any pit-fall if water was provided during acclimatization. Therefore, crabs were tested for attraction to water vapour from distilled water (10 ml) and ASW (10 ml), in addition to the food odours from banana (10 g), overripe banana (10 g), salmon (10 g), ground peanut snacks (2 g) (for details, see electronic supplementary material). Here, an empty cup was used as control. As negative control, two empty cups were used. Furthermore, ground peanut snacks were tested in combination with distilled water using distilled water as control. Both cups in a setup were always covered with perforated lids to avoid visual stimuli, while allowing odour stimuli. Banana extract from headspace collection of volatile odours from banana was tested at a concentration analogous to *ca* 10 g banana dissolved in 100 µl mineral oil (for details, see electronic supplementary material). The banana extract was added to 1.5 ml glass vials, subsequently placed open in the small plastic cups in the pit-falls. The banana extract was tested versus a control vial containing 100 µl mineral oil (with compensational amount solvent, dichloromethane, as in the banana extract; for details, see electronic supplementary material). In addition, the banana extract was tested in combination with distilled water (10 ml; added into the small plastic cups next to odour and control vials).

Some representative synthetic compounds that had been found to be physiologically active in the EAG experiments were tested in the bioassay, i.e. propionic acid, propanal, triethylamine and propylamine. Although defined as physiologically inactive (see §3), isoamyl acetate was tested as a, for humans, very typical banana odour. All compounds were tested at 10^−2^ dilutions in mineral oil, prepared just before use in the bioassay. A total of 500 µl of the test solution was added to 1.5 ml glass vials, subsequently placed open in the small plastic cups of the pit-falls. Mineral oil (500 µl) served as control. Additionally, in order to increase the humidity during stimulus detection, all compounds were tested in combination with distilled water (10 ml; added into the plastic cup next to the vial). Relevant test concentration for these tests was determined in a pilot test, testing triethylamine at 10^−4^, 10^−3^, 10^−2^ and 10^−1^ dilutions, and hereby finding significant responses at 10^−2^ when tested with water and only at 10^−1^ when tested without water. To check the reliability of the crab behaviour, tests with highly attractive food (banana) were repeated regularly throughout the test period.

### (e) Data processing and statistics

We compared the chemical properties of all compounds screened for EAG activity by using a multivariate principal components analysis (PCA) of their location in a multi-dimensional physico-chemical odour space. In such odour space, odourants that are structurally similar map close together, while odourants that are dissimilar map far apart. To construct the physico-chemical odour space, the molecular structure for each odourant was obtained from PubChem (http://pubchem.ncbi.nlm.nih.gov/search/) and was put into DRAGON v. 5.5 2007 for Windows (Talete, srl; http://www.talete.mi.it/), providing 3224 molecular descriptors for each odourant, such as, e.g. molecular weight, functional group and carbon chain length [21]. Descriptors were normalized by dividing the value of each descriptor by the mean value across all odourants: normalized descriptor = descriptor/mean value. Descriptors with only 0 values were removed resulting in 1474 descriptors in the final odour-descriptor matrix further analysed. PCAs were performed with PAST (http://folk.uio.no/ohammer/past/). To test if compounds that generate the same type of EAG response cluster in odour space, we performed a one-way analysis of similarities (AnoSim) with PAST to test for differences in Euclidean distance between active and inactive compounds and between compounds yielding negative and positive EAG responses.

We also compared the water solubility of physiologically active and inactive compounds determined for *C. clypeatus*. Solubility data (log mg l^−1^) for each compound was obtained from ChemIDplus Lite (http://chem.sis.nlm.nih.gov/chemidplus/chemidlite.jsp) when available (otherwise left out of the analysis), *n* = 35 and 91 for active and inactive compounds, respectively. Difference in water solubility between active and inactive compounds was tested with a Mann–Whitney U-test, performed with SPSS, v. 17.0 (SPSS Inc.).

To test whether humidity influences EAG response in *C. clypeatus* and *D. melanogaster*, a repeated measurement ANOVA was used, followed by a Tukey–Kramer multiple comparisons test, performed with InStat, v. 3.06 for Windows (GraphPad Software Inc.).

For statistical analysis of the two-choice bioassay, the distribution of crabs between pit-falls with test odourant and control was tested for deviation from random (1 : 1) distribution, using a *G*-test with Williams's correction to compensate for increased type I error. The samples from tests from different nights were pooled.

## 3. Results

### (a) Physiologically active compounds

**Figure 1.**
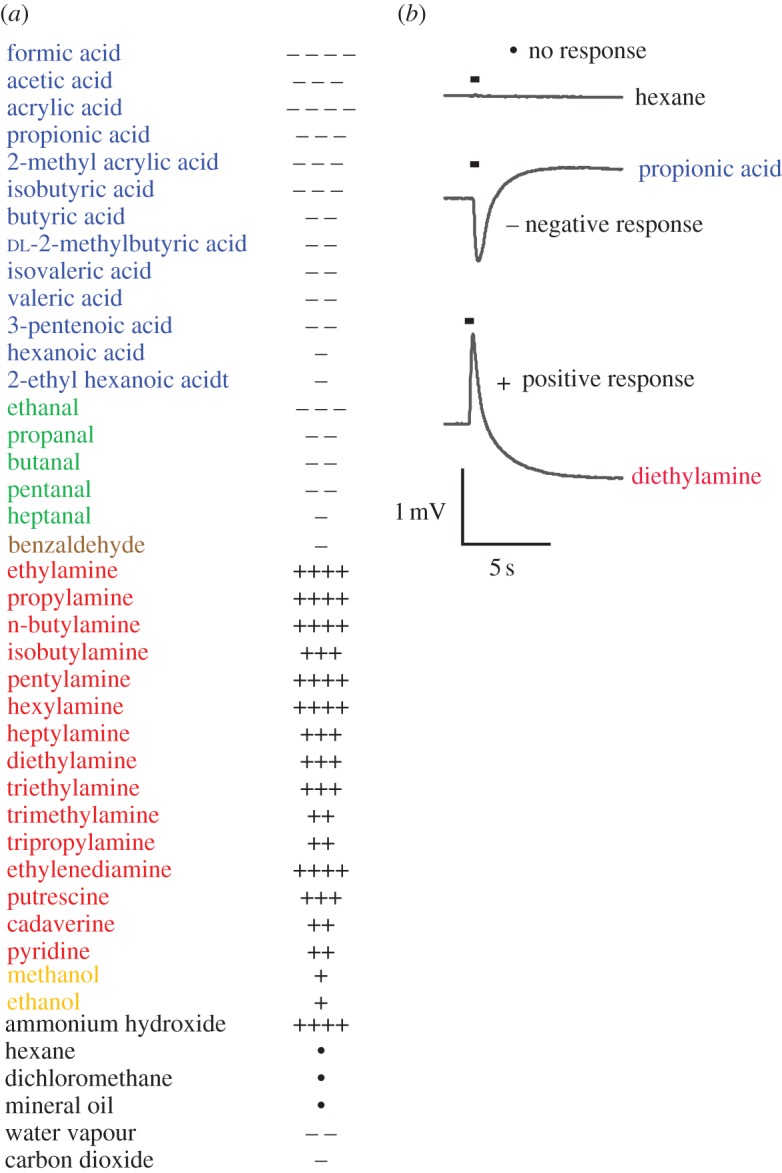
Electroantennogram (EAG) responses in *Coenobita clypeatus.* (*a*) Active odourants elicited a negative (**−**) or a positive (**+**) EAG response. Stimuli were presented at 10^−1^ dilutions, except the solvents hexane, dichloromethane, mineral oil and water that were presented pure, and carbon dioxide that was presented by extracting headspace over dry ice with a syringe and injecting this headspace into the constant airstream passing over the antennule. Responses were calculated by measuring the maximum response peak (negative or positive) following the 0.5 s odourant presentation and subtracting the response to pure solvent. For the solvents (hexane, dichloromethane, mineral oil and water), responses to a blank were subtracted, and for carbon dioxide, an equivalent air volume served as control. Symbols represent the average response (*R*) of at least 10 antennular preparations. Dots (•), no response: −30 µV < *R* < 30 µV; one minus symbol, −30 µV ≥ *R* > −100 µV; two minus symbols, −100 µV ≥ *R* > − 1000 µV; three minus symbols, −1000 µV ≥ *R* > −5000 µV; four minus symbols, *R* ≤ −5000 µV; one plus symbol, 30 µV ≤ *R* < 100 µV; two plus symbols, 100 µV ≤ *R* < 1000 µV; three plus symbols, 1000 µV ≤ *R* < 5000 µV; four plus symbols, *R* ≥ 5000 µV. Compounds are colour coded according to functional group: blue, acid; green, aldehyde; brown, aromatic; red, amine; orange, alcohol. (*b*) Examples of EAG responses to 10^−1^ odourant dilutions*,* showing typical negative and positive responses to a carboxylic acid (propionic acid) and an amine (diethylamine). In the upper control trace, the pure solvent (hexane) served as stimulus (stimulus presentation indicated by bar above each trace).

The results from the broad screening of compounds for EAG activity in *C. clypeatus* established physiological activity for several short-chained carboxylic acids and aldehydes, several mono- and diamines, as well as to methanol, ethanol, benzaldehyde, ammonium hydroxide and water vapour (figure 1*a*). Odourants from the other chemical classes, i.e. esters, lactones, ketones, aromatics (besides benzaldehyde), cyclic terpenes, ethers, furans, sulphur compounds, thiocyanates and amino acids, were not physiologically active (for inactive compounds, see electronic supplementary material). Carbon dioxide was scored as EAG-active, having an average response of −131 µV (i.e. less than or equal to −30 µV), but activity was only noted in two out of 10 preparations and must therefore be considered unreliable. The active compounds could be grouped into two categories, where acids, aldehydes and water vapour generate negative EAG responses, while amines, ammonium hydroxide and alcohols generate inverted, positive EAG responses (figure 1*a,b*).

To visualize the representation of all compounds screened for EAG response in *C. clypeatus* in a multi-dimensional physico-chemical odour space, we projected it into two dimensions via a PCA (see electronic supplementary material, figure S1*a*). The investigated compounds cover a large part of odour space, with no significant separation of physiologically active and inactive compounds (one-way AnoSim for Euclidean distances, *p* = 0.8). Within the active odourants, however, compounds eliciting negative and positive EAG responses were clearly separated in their odour space representation (one-way AnoSim for Euclidean distances, *p* < 0.0001; electronic supplementary material, figure S1*b*), demonstrating that they differ in chemical properties.

As water vapour greatly influenced the physiological and behavioural responses (see §3*b*,*d*,*e*), we checked for the water solubility of all compounds screened for EAG activity in *C. clypeatus*. Here, we found a highly significant difference in water solubility, where physiologically active compounds were found to be more water-soluble, whereas inactive compounds can be of any solubility (Mann–Whitney U-test, *Z* = −4.58, *p* < 0.001; electronic supplementary material, figure S2).

**Figure 2.**
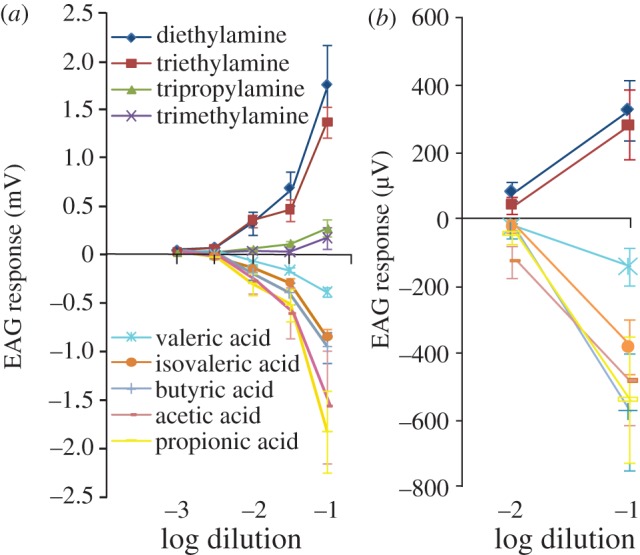
Dose-dependent EAG responses to selected odourants (*a*) in the land hermit crabs *Coenobita clypeatus* and (*b*) in the marine hermit crab *Pagurus bernhardus*. Odourants (1 µl) were presented to the antennule in ascending steps. For line colours and markers in (*b*), see (*a*) (error bars represent s.e.m.; *n* = 9–14 in (*a*); *n* = 5 for the lower and 10 for the higher concentration in (*b*)).

The EAG responses to acids and amines were clearly dose-dependent in *C. clypeatus* (figure 2*a*). Relatively high thresholds were noted, with responses from 10^−2^ dilution (equivalent to 7.5–9.4 µg stimulus load) for isovaleric and valeric acid and trimethylamine, whereas triethylamine and butyric, propionic and acetic acid were active at 1 : 500 dilutions (1.5–2.1 µg stimulus load) and diethylamine already at 10^−3^ dilution (0.7 µg stimulus load). Also *C. compressus* showed dose-dependent responses to these odourants although the magnitude of the responses was about 10-fold lower (see electronic supplementary material, figure S3). Furthermore, the reversed EAG response pattern for acids and amines held true also in this species, and notably also in the marine hermit crab *P. bernhardus* (figure 2*b*).

### (b) Humidity level greatly affects the physiological response in *Coenobita clypeatus*

**Figure 3.**
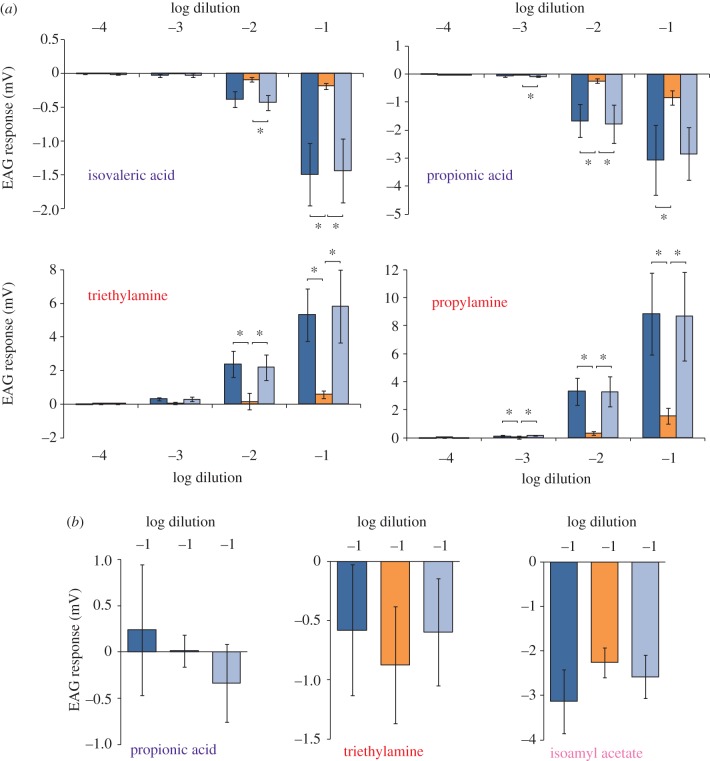
EAG response to odourants tested at different humidity levels, for *Coenobita clypeatus* (*a*) and *Drosophila melanogaster* (*b*). A first recording session, including the odourants and concentrations to be tested, was performed at the initial high humidity (dark blue, 86–91% RH), a second at the low humidity (orange, 32–35% RH) and finally, a third at the high humidity level (final high humidity—light blue, 86–91% RH). (*a*) For *C. clypeatus*, four odourants were presented at dilutions from 10^−4^ to 10^−1^ in each recording session (*n* = 5, error bars represent s.e.m. asterisk (*), *p* < 0.05-values based on repeated measurement ANOVA, followed by Tukey–Kramer multiple comparisons test). (*b*) For *D. melanogaster*, three odourants were presented at 10^−1^ dilution in a recording session (no significant differences between responses at the different humidity levels, tested with repeated measurement ANOVA; error bars represent s.e.m., *n* = 6).

Motivated by increased behavioural responses when water was added to the stimuli (see §3*d*,*e*), we repeated EAG recordings under different humidity levels. Our results confirm the importance of water vapour for olfactory performance in *C. clypeatus*: At 10^−1^ and 10^−2^ dilutions, the EAG responses to all tested odourants were significantly larger at high humidity as compared with responses at low humidity (three up to more than 10 times larger depending on odourant and concentration tested; figure 3*a*) while at 10^−3^ dilutions, humidity significantly affected the EAG response only for propionic acid, triethylamine and propylamine (figure 3*a*). Since 10^−4^ dilutions did not elicit EAG activity (i.e. mean response was never greater than 30 µV or less than or equal to 30 µV), this concentration was not analysed. Generally, the EAG response recovered and was as high at a repeated second high humidity test as it had been during the initial high humidity recording, confirming that the decrease in response at the low humidity level was not due to a deteriorating antennular preparation. In contrast, in the vinegar fly *D. melanogaster*, no difference in EAG response was found at different humidity levels (figure 3*b*).

None of the compounds defined as EAG inactive in the initial screening experiment were found active when re-tested at higher humidity, which confirms that the inactivity was not due to a too low humidity level (for compounds, see electronic supplementary material).

### (c) Behavioural response to odours from food and water

**Figure 4.**
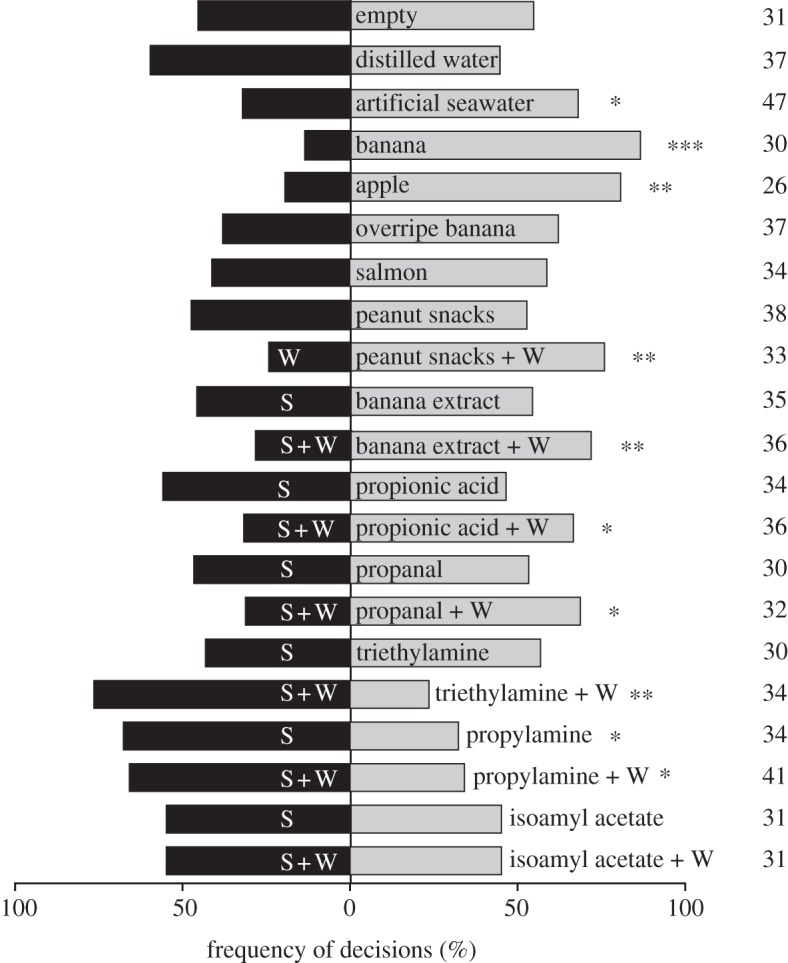
Behavioural response of *C. clypeatus* to natural and synthetic odourants in a two-choice bioassay. Different natural and synthetic odourants (light grey, crabs to treatment; with and without 10 ml distilled water, W) were tested against an empty control, water and/or solvent (S) (black, crabs to control). Two empty controls were used as negative control (top bars). Synthetic odourants were presented at 10^−2^ dilutions in mineral oil. (*G*-test, * *p* < 0.05, ** *p* < 0.01, *** *p* < 0.001, *n* indicated to the right for each test.)

In nature, *Coenobita* spp. use odours to find food [7,8]. By establishing a reliable two-choice bioassay in the laboratory, we wanted to repeat these experiments under more controlled conditions. We could show that *C. clypeatus* is significantly attracted to natural, complex odours from fruit and seawater (figure 4). While the distribution of crabs between two empty pit-fall traps was random, almost all crabs (26 out of 30, i.e. 87%) selected a trap with banana over an empty trap and similarly, 21 out of 26 (81%) chose the apple treatment over an empty control. However, the crabs were not attracted to odours from all food items provided, since neither overripe banana nor raw salmon were chosen over the controls. Furthermore, the crabs were significantly attracted to ASW, but not to distilled water.

### (d) Water vapour greatly affects the behavioural response to natural, complex odours

To test the hypothesis that water vapour influences the attractiveness of natural, complex food odours, we used ground peanut snacks, being a dry food item that is highly preferred by the crabs when provided during rearing, as well as banana extract. When tested as a dry stimulus, *C. clypeatus* was not attracted to peanut snacks, but when presented in combination with water, this food item was significantly attractive to the crabs (figure 4). Similarly, banana extract was found to be significantly attractive to the crabs only when presented in combination with water (figure 4).

### (e) Behavioural response to physiologically active odourants

To test if the crabs also respond behaviourally to physiologically active odours, some representative synthetic compounds were tested in the bioassay. When tested as pure stimuli, neither propionic acid, the aldehyde propanal nor triethylamine elicited a behavioural reaction in the crabs, but propylamine was significantly avoided (figure 4). However, when tested in combination with water, both amines were significantly repulsive, while both propionic acid and propanal were significantly attractive to the crabs (figure 4). In line with the physiological results, isoamyl acetate did not elicit any behavioural response, neither when tested alone, nor in combination with water (figure 4).

## 4. Discussion

The new selection pressures that come with the transition from aquatic to terrestrial life obviously have great impact on olfactory function and abilities. Previous behavioural field work on *Coenobita* spp. has shown that the conquest of land has led to adaptations of the olfactory system that enable these omnivorous crustaceans to localize food, water and shell resources [7,8,10,11]. Our behavioural experiment under laboratory conditions confirms these findings, demonstrating that *C. clypeatus* clearly detects volatiles from fruit and seawater and uses these cues to locate a target. Furthermore, based on olfactory cues, the crabs seem to estimate food quality, as the smelly cues salmon and overripe banana did not elicit significant responses (figure 4). Thus, even though additional orientation mechanisms such as celestial compass, vision and wind direction may be used for navigation (reviewed by Greenaway [4]), olfaction certainly plays a prominent role in the foraging behaviour of terrestrial hermit crabs. However, we know little of how land-living crustaceans have solved the task of odour detection. Here, we established biologically active odourants in the terrestrial hermit crab *C. clypeatus*, and furthermore show that humidity is most critical for the function of the crab's olfactory sense.

### (a) Physiologically active compounds

Obviously, the synthetic odourants screened for activity in *C. clypeatus* only represent a fraction of all potentially relevant odourants for such an omnivorous species and thus, it is likely that key ligands have been overlooked. Nevertheless, the compounds investigated represent compounds with a relatively high degree of chemical diversity (see electronic supplementary material, figure S1*a*). Only compounds that are at least slightly water-soluble were found to be physiologically active (see electronic supplementary material, figure S2). It is important to remember that although living their adult life on land, *Coenobita* go through several planktonic larval phases in the sea before emerging to land as a megalopa. During these marine stages, chemical cues may be involved in, for example, foraging, finding suitable habitats and shells and avoiding predators, since this has been established in other crustacean taxa [22,23]. Our findings suggest that although specific morphological adaptations, such as ultrastructural features of the aesthetascs, are required to cope with desiccation stress associated with life on land, the adult olfactory system may very well operate with the same chemosensory ligands as during the juvenile stages. This is further supported by our finding that even the marine hermit crab, *P. bernhardus*, was able to detect volatile carboxylic acids and amines in the—in air performed—EAG experiments (figure 2*b*). Also the marine hermit crab *Clibanarius vittatus* has been shown to react to volatile compounds, where seawater containing volatiles from conspecific haemolymph stimulate shell investigation behaviour [24]. Olfactory responses that were conserved during the switch from an aquatic to a terrestrial lifestyle have been shown in the tiger salamander *Ambystoma tigrinum*, where airborne volatiles, volatile solution and amino acid solutions activated the olfactory epithelium in both aquatic larvae and terrestrial adults [25].

Most interestingly, the identified physiologically active odourants in *C. clypeatus*, but also those in the land-living *H. reaumuri* [20], agree more or less perfectly with the chemical groups that so far have been identified as ligands for the ionotropic receptors (IRs), the recently identified family of ionotropic glutamate receptors that has been shown to be chemosensory in *D. melanogaster* [26,27]. IRs and IR-related genes have so far been identified in chemosensory organs of animals as diverse as arthropods, nematodes and molluscs, including the two crustaceans water flea *Daphnia pulex* and lobster *Homarus americanus* [26,28,29]. Contrary to insects, that in addition express another receptor family, the so-called olfactory receptors (ORs), sequencing the genome of *D. pulex* did not reveal any genes similar to the insect OR genes [30], whereas 85 IRs were identified [28]. The molecular identity of chemosensory receptors in the antennules is unknown for *C. clypeatus*. However, our data suggest that olfaction in these crustaceans is mainly accomplished via a set of IRs, although other still unidentified gene families cannot be excluded. This agrees with the hypothesis that the IRs are a more ancient form of chemosensory receptors, as compared with the insect OR family that would be a relatively recently expanded gene lineage concomitant of evolution of terrestriality in the insect or their hexapod ancestors [26,28].

The EAG responses to acids and amines were clearly dose-dependent in the two *Coenobita* species (figure 2*a*; electronic supplementary material, figure S3). Besides some difference in magnitude, the response profiles of *C. clypeatus* and *C. compressus* were analogous and several of the compounds found to be inactive in *C. clypeatus* were inactive also in the semi-terrestrial *C. compressus* (data not shown). The relatively high thresholds (*ca* 1–10 µg stimulus load) for active compounds suggest a less sensitive olfactory system as compared with the most sensitive odourant receptors (ORs) in insects [31]. However, the difference in the vapour pressure between different chemical compounds makes direct comparison difficult. Surprisingly, the response profile for these two *Coenobita* species differ substantially from previous findings in the closely related Coenobitidae *B. latro* [9]. Notably, the compounds active in *B. latro* are insoluble or only slightly soluble in water, as opposed to active compounds in *C. clypeatus*. Detection limits in *Coenobita* are in line with those for *B. latro*, besides dimethyl trisulphide and dimethyl disulphide to which *B. latro* was much more sensitive [9]. The low number of compounds tested in *B. latro,* as well as the exclusion of all chemical groups found to be active in the genus *Coenobita*, makes comparison difficult. Nevertheless, the different findings in these species suggest that the functional characteristics of the peripheral olfactory system differ substantially between *Coenobita* and *B. latro. Birgus latro* seems to have developed a higher capacity to detect airborne volatiles when compared with *Coenobita* and may even have additional odour receptor groups present. Thus, compared with the system of *Coenobita*, the olfactory system of *B. latro* seems to be more similar to that of insects [5,9].

### (b) Odourants with different chemical properties elicit reversed response patterns

Our results reveal two groups of compounds, eliciting reversed EAG responses (figures 1 and 2). These groups were also found to differ significantly in chemical properties, shown by their separation in physico-chemical odour space representation (electronic supplementary material, figure S1*b*). The opposite EAG responses can be interpreted as odourants activating different pathways involved in signalling transduction. In marine lobsters (*Panulirus argus*), odours may depolarize or hyperpolarize olfactory sensory neurons (OSNs) and different pathways are directly linked to opposing outputs [32–34]. Excitatory and inhibitory receptor potentials coexist in the same lobster OSN and a given odourant can excite and inhibit different OSNs [32,33]. In *C. clypeatus*, water vapour and possibly carbon dioxide show a negative EAG response. Contrary to our findings, in *B. latro*, inverted polarization of the EAG response was induced by water vapour, when compared with the odourants tested and the very pronounced response to carbon dioxide found in this species [9].

Interestingly, our findings suggest that odourants eliciting similar physiological response relate also to behavioural responses: the carboxylic acid and the aldehyde that elicited negative EAG responses, were both significantly attractive to the crabs, whereas the two amines that elicited inverted, positive EAG responses, were both significantly repulsive to the crabs (figure 4). However, to test whether this is a global feature, further comparison of behavioural responses among chemically similar odours is needed. The behavioural response to amines agrees with results from several terrestrial species, where amine odourants generally signal the presence of spoiled food and elicit avoidance reactions [35], in contrast to aquatic environments, where amines function as feeding cues [1]. Acids on the other hand are often associated with avoidance behaviour in terrestrial insects [36], but may also function attractants, for example, the mosquito *Aedes aegypti* [37] and *D. melanogaster* [38].

### (c) Water vapour greatly influence olfactory functions

*Coenobita* clearly detects water vapour as shown by the EAG response, but in agreement with Vannini & Ferretti's [11] study on *Coenobita rugosus* and *C. cavipes*, we found that also in *C. clypeatus*, water vapour alone does not elicit behavioural responses, since crabs were not attracted to distilled water even after 24 h water deprivation (figure 4). Our results demonstrate, however, that although not attractive by itself, water vapour is most critical for complex food odours, as well as individual, synthetic compounds, to induce behavioural response in *C. clypeatus* (figure 4). It is possible that additional sensation of water vapour is needed to make an odour relevant, since in nature, supposedly mostly moist food sources occur. However, given that the amines induced repulsion also without water at higher concentrations instead suggest less efficient odour detection at low humidities. The additional water next to the bait in our bioassays would thus increase the humidity level sufficiently to assist odour detection. For *C. clypeatus*, this hypothesis is strongly supported by the fact that also the physiological response is greatly influenced by humidity, with much greater EAG responses at higher humidity levels (figure 3*a*). Possibly, water vapour is needed for odourant transmission and/or the diffusion of the odourant through the permeable, thin-walled aesthetasc surface into the lumen of the sensilla. How odourants are transported into the aesthetasc sensilla is however unknown in *Coenobita*. In contrast to insects, there is no evidence of distinct pores in crustacean olfactory sensilla (aesthetascs) [17], but dye studies in *C. compressus* suggest that the cuticule of the distal region of the aesthetasc is permeable to aqueous solutes [19]. In contrast, no effect of humidity was found on EAG recordings in *D. melanogaster* (figure 3*b*), which indicates that this is not a general feature in arthropods. The humidity dependence of the crab's olfactory system possibly relates to the relatively short evolutionary history on land of *C. clypeatus.* Future studies shall reveal, whether the independence of humidity as observed in *D. melanogaster* is a general feature in and/or restricted to insects. Interesting candidates for these studies would on the one hand be the completely desert-adapted crustacean *H. reaumuri*, and on the other hand an insect that is closely bound to water, like the water strider *Gerris remigis*.

In conclusion, it seems that although terrestrial hermit crabs have evolved a sense of smell evidently functional on land, their chemosensory system is only partly adapted to the terrestrial environment, with a preserved general design of aquatic crustaceans. We found that the *Coenobita* olfactory system is restricted to a limited number of mainly water-soluble molecules as olfactory stimuli, from chemical classes known to be ligands for the IRs [26], but also known to stimulate foraging in aquatic crustaceans. Furthermore, our results reveal that water vapour is most critical for both physiological and behavioural olfactory function in *C. clypeatus*. Coenobitidae are dependent on closeness to the sea for reproduction and their distribution is restricted to the coast or small islands in tropical/subtropical areas [3,4]. Our results suggest that besides restricting desiccation, the high humidity of these areas is also essential for efficient odour detection in these terrestrial hermit crabs.
